# Machine Learning Prediction Model for Inflammatory Bowel Disease Based on Laboratory Markers. Working Model in a Discovery Cohort Study

**DOI:** 10.3390/jcm10204745

**Published:** 2021-10-16

**Authors:** Sebastian Kraszewski, Witold Szczurek, Julia Szymczak, Monika Reguła, Katarzyna Neubauer

**Affiliations:** 1 Department of Biomedical Engineering, Wroclaw University of Science and Technology, Pl. Grunwaldzki 13, 50-377 Wroclaw, Poland; 2 Doctoral School, Wroclaw University of Science and Technology, Wybrzeze Wyspianskiego 27, 50-370 Wroclaw, Poland; witold.szczurek@pwr.edu.pl; 3 Faculty of Fundamental Problems of Technology, Wroclaw University of Science and Technology, Wybrzeze Wyspianskiego 27, 50-370 Wroclaw, Poland; 236405@student.pwr.edu.pl (J.S.); 236689@student.pwr.pl (M.R.); 4 Divison of Dietetics, Department of Gastroenterology and Hepatology, Wroclaw Medical University, Borowska 213, 50-556 Wrocław, Poland

**Keywords:** inflammatory bowel disease, ulcerative colitis, Crohn’s disease, artificial intelligence, machine learning, model, prediction

## Abstract

Inflammatory bowel disease (IBD) is a chronic, incurable disease involving the gastrointestinal tract. It is characterized by complex, unclear pathogenesis, increased prevalence worldwide, and a wide spectrum of extraintestinal manifestations and comorbidities. Recognition of IBD remains challenging and delays in disease diagnosis still poses a significant clinical problem as it negatively impacts disease outcome. The main diagnostic tool in IBD continues to be invasive endoscopy. We aimed to create an IBD machine learning prediction model based on routinely performed blood, urine, and fecal tests. Based on historical patients’ data (702 medical records: 319 records from 180 patients with ulcerative colitis (UC) and 383 records from 192 patients with Crohn’s disease (CD)), and using a few simple machine learning classificators, we optimized necessary hyperparameters in order to get reliable few-features prediction models separately for CD and UC. Most robust classificators belonging to the random forest family obtained 97% and 91% mean average precision for CD and UC, respectively. For comparison, the commonly used one-parameter approach based on the C-reactive protein (CRP) level demonstrated only 81% and 61% average precision for CD and UC, respectively. Results of our study suggest that machine learning prediction models based on basic blood, urine, and fecal markers may with high accuracy support the diagnosis of IBD. However, the test requires validation in a prospective cohort.

## 1. Introduction

Inflammatory bowel disease (IBD) is a chronic, incurable disease of the gastrointestinal tract represented by two most common forms: ulcerative colitis (UC) and Crohn’s disease (CD). The pathogenesis of IBD is not fully explained, and according to the “IBD interactome” concept, it involves interrelation between genetic, microbiological, environmental, and immune factors [1,2]. Though unclear pathogenesis results in the lack of effective therapeutic modalities and the absence of efficient prevention, this in the light of the increasing prevalence of IBD worldwide has a particular meaning. Consecutively, there is no single test sufficient to provide a diagnosis. Recognition of IBD is based on a combination of clinical symptoms, laboratory tests, and endoscopic and imaging tests together with pathological examination [3,4]. Remarkably, despite progress in endoscopic and imaging techniques, the delayed diagnosis of IBD remains a significant clinical problem due to the lack of effective non-invasive diagnostic tools that can be performed by a primary care physician (internists or general practitioners (GP)). Further, in case of Crohn’s disease, concomitant diseases, and older age were identified as key reasons for the delayed IBD diagnosis [5]. At the same time, the age profile of IBD patients is changing and there is an increase in early-onset and late-onset IBD prevalence. Both groups (older adults, which frequently suffer from various comorbidities, as well as children) would particularly benefit from the non-invasive diagnostic test. However, colonoscopy remains a gold standard in IBD diagnosis, monitoring of the disease course, and response to the therapy, as well as colorectal cancer screening [6,7,8,9]. Still, despite its obvious advantages, it is highly invasive, expensive, time-consuming, requires qualified medical personnel and patient‘s preparation, and is often poorly tolerated by patients themselves. Besides, in the pandemic all low-contact medical procedures are preferred. Therefore, a simple diagnostic methodology based only on markers from blood, urine and stool that can be performed by a GP would be imperative in the early diagnosis of IBD.

Taken together, increased IBD incidence worldwide, increased incidence both among children and older adults, new therapeutic goals in IBD, and drawbacks of endoscopy demonstrate an urgent need for searching for the non-invasive biomarker or panel of biomarkers in IBD. Moreover, the recent COVID-19 pandemic not only emphasized the advantages of the non-invasive point of care tests but also demonstrated the advantages of novel technologies [10,11].

Aims of our study were:

1. building the machine learning model based on routinely performed laboratory blood, urine, and fecal tests to support differentiation between IBD patients and non-IBD patients
2. comparison of the effectiveness of our model to standard inflammatory serum marker, that is C-reactive protein (CRP), in the prediction of IBD, creating a website-based application supporting the prediction of the presence of IBD

## 2. Materials and Methods

Data of 702 records of 372 consecutive polish patients from the Lower Silesia region with inflammatory bowel disease hospitalized in the Department of Gastroenterology and Hepatology, Wroclaw Medical University between 2019 and 2020 due to the disease diagnosis, disease flare, and therapy or monitoring of disease course were analyzed. All historical patients’ data were collected with the consent of the Bioethics Committee of the Wroclaw Medical University Nº KB-504/2021. More precisely, the following were used: 319 records from 180 patients with ulcerative colitis and 383 records from 192 patients with Crohn’s disease. The control group was composed of 315 records from 271 patients with non-inflammatory and non-malignant bowel diseases, i.e., patients with uncomplicated diverticular disease, patients with benign colonic polyps, and patients after colonic polypectomy admitted for surveillance colonoscopy. The control group patients were chosen to be without leading associated diseases. All patients were of Caucasian ethnicity. Patients classification and baseline characteristics of the studied groups are presented in Table 1 and Table 2. Control groups were chosen to be as much as possible similar to the study group in order to let the algorithm automatically find the fine balance in relationship between laboratory markers invisible at first glance, and in order to allow the generalization of our model in that manner.

Each patient chart was characterized by a breakdown of anonymized personal information, detailed results of tests performed, and medical record. By means of data anonymization, the extracts obtained for analysis did not contain such information as name, surname, personal identity number, place of residence, or numbers in the hospital ward book. Between physical patients’ parameters, only patient age and gender were available in the study parameters. However, the medical record contained all information about laboratory tests performed to date, medical procedures applied, consultations performed, or medications administered.

The input data was initially divided into learning and test sets with a 7:3 ratio due to small sample size. Then, scaling and dimensionality reduction of the data were performed using the principal component analysis (PCA) method. About 443 primary extracted features were reduced to 64 unique instances since the datasets differed in incomplete data threshold. Most of the samples in all groups (UC-study, CD-study, or control) were not complete, so the obtained data were also subjected to data supplementation by multiple imputation method using IterativeImputer from the Scikit-learn library [12]. Specifically, for each feature (physical or laboratory marker, as shown in Table 3), the median was calculated on the observed data, which was decreased or increased by a random value from the interval <−SD, +SD> (where SD is the standard deviation calculated on the observed samples) and then iteratively entered in each cell with the missing value. Such an operation was performed separately for each feature and separately for the studied and control groups. Exceptions are values that have been converted from text data. For such data, an integer is drawn from the given range with appropriate weights, which were calculated by dividing the number of occurrences of unique values by the total number of feature values.

In the first step, machine learning (ML) classifiers with high prediction were run through hyperparameters tuning. These classifiers included logistic regression, k-nearest neighbor, decision tree forests, support vector machine, and gradient boosting, with different hyperparameters. Next, a majority voting classifier was introduced for each set, based on ensemble learning, which combines several models to create a single most optimal predictive model. Automatic tuning of hyperparameters was performed using the grid method with greedy algorithm. The algorithm greedily selects the features that give it the best result. It checked between 20 and 10 featurings. Each classifier can thus have optimally chosen the number of features that gives the best result. The whole process was accompanied by cross-validation with a **k** parameter equal to 10, which was proven to give the best variance to load trade-off.

### Evaluating the Effectiveness of the Model

In order to correctly select classifiers, a mechanism for estimating performance had to be provided. Without introducing a way to evaluate the model, it would not be possible to determine the optimal balance between variance and load. Every model is exposed to the risk of under-fitting (i.e., high loading) due to low complexity or over-training (i.e., high variance due to too high complexity).

## 3. Results

### 3.1. Data Filtering and Input Features

The processing operation of the provided data resulted in 443 data features, which represented the unique biological or blood-, urine-, or stool-based laboratory parameter of the patient. Unfortunately, most of these laboratory tests were performed on few patients only and do not represent a reliable source of information by their presence at less than 30% in a given group (UC-study, CD-study, or control). Filtering these data ultimately yielded 64 features present in all groups (Table 3), from which the data analysis was then performed. It should also be noted that in addition to the total number of white blood cells, morphological studies also report the expanded blood pattern, that is, the number of each type of white blood cell per unit volume. The duplicate feature names are not an error but a distinction, since for each total blood cell count (indicated in Table 3 with the ‘#’ sign), a percentage (‘%’ sign in Table 3) within the expanded blood pattern has also been calculated. 

### 3.2. Machine Learning Classifiers

Each machine learning task is to work out a certain solution with the help of an appropriate mathematical model, whose parameters are not known at the beginning. At the input of the model, we have data which are the specific values of certain features, while at the output of the model we get the solution to the task associated with certain domain objects as individuals, examples, specimens, measurements in the world. Here, the features are laboratory markers obtained from historical patients. The problem of machine learning is the automatic, machine building of the model with the help of an appropriate algorithm. Here the following classification algorithms were used.

#### 3.2.1. Logistic Regression

**Figure 1:**
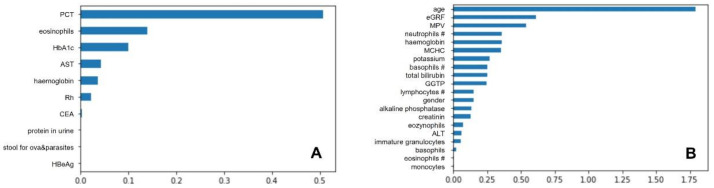
Logistic regression features importance for ulcerative colitis (**A**) and Crohn’s disease (**B**). Abbreviations for (**A**): PCT, plateletcrit; AST, aspartate aminotransferase; CEA, carcinoembryonic antigen; HBeAg, Hepatitis B e Antigen; abbreviations (**B**): eGFR, estimated glomerular filtration rate; MPV, mean platelet volume; MCHC, mean corpuscular haemoglobin concentration; GGTP, gamma-glutamyl transpeptidase; ALT, alanine transferase.

For the set of classifiers, multiple variants of logistic regression were tested for optimization and to find the best combinations of parameters such as optimization algorithm, fitting method, weights, regularization penalties, and the so-called C parameter. The grid method showed that the worst prediction results were achieved by classifiers with the regularization set to L1 (internal algorithm setting under Python programing language) and the inverse of the regularization strength equal to 0.01. No significant changes were observed between the default value of C and 0.1, and finally C value of 0.1 was considered the optimal value. Different values of the optimization algorithm, by themselves, did not affect prediction performance, suggesting their importance only when combined with other parameters. Models with a linear optimization algorithm and both variants of the fitting method received good results. Finally, the following hyperparameters were found to be the optimal for UC dataset: {‘classifier__C’: 0.01, ‘classifier__penalty’: ‘l2’, ‘classifier__solver’: ‘newton-cg’, ‘featureSelector_ _n_features_to_select’: 10}, and the most relevant were following features: plateletcrit (PCT), eosinophils, haemoglobin A1c (HbA1c), aspartate aminotransferase (AST), haemoglobin, Rhesus (Rh), carcinoembryonic antigen (CEA), protein in urine, microscopic stool for ova and parasites test, and Hepatitis B e Antigen (HBeAg). Meanwhile, for CD dataset the most optimal clasificator parameters were {‘classifier__C’: 0.1, ‘classifier__penalty’: ‘l2’, ‘classifier__solver’: ‘newton-cg’, ‘featureSelector_ _n_features_to_select’: 20}, resulting in the following important features: age, estimated glomerular filtration rate (eGRF), mean platelet volume (MPV), neutrophils count, haemoglobin, mean corpuscular haemoglobin concentration (MCHC), potassium, basophils count, total bilirubin, gamma-glutamyl transpeptidase (GGTP), lymphocytes count, gender, alkaline phosphatase, creatinine, eosinophils, alanine aminotransferase (ALT), immature granulocytes, basophils, eosinophils count, and monocytes. Logistic regression features of importance are presented in Figure 1.

#### 3.2.2. The k-Nearest Neighbor

The nearest neighbor algorithm gave different results depending on the completed data, but the given results never deviated significantly from the other classifiers. On the other hand, for changing the parameters there were clear differences between the prediction results. To optimize the classifier, parameters such as the number of nearest neighbors, *p*-value, computational algorithm, and weights were checked. The default value of 5 was considered as the optimal value of nearest neighbors, due to the fact that for other values of the parameter, the prediction performance decreased. The *p*-value corresponds to the metric used, and when changing to the Manhattan metric, a slight degradation in performance was noted. Therefore, the Euclidean metric was considered the optimal metric. For these default parameters, changing the computational algorithm and weights had no significant effect. The grid method did not show significant differences in prediction depending on the different optimization algorithms or distance metrics used. However, they had significantly higher prediction accuracy (on the order of 82–85%) than for classifiers with a single parameter for the number of neighbors. Finally, the following hyperparameters were found to be the optimal for UC dataset: {‘classifier__n_neighbors’: 12, ‘featureSelector__n_features_to_select’: 13}, and the most relevant were following features: AST, HBe Ag, microscopic stool ova and parasites test, protein in urine, glucose in urine, blood in urine, erythrocytes in urine, erythroblasts, lymphocytes, MPV, neutrophiles, PLT, and age. Meanwhile for CD dataset the most optimal clasificator parameters were {‘classifier__n_neighbors’: 18, ‘featureSelector_ _n_features_to_select’: 17}, resulting in the following important features: total bilirubin, CRP, GGTP, creatinine, basophils, eosinophils, erythroblast, HCT, haemoglobin, lymphocytes, monocytes, and immature granulocytes.

In the nearest neighbor algorithm exact features importance is not possible to extract, as it is for other classifiers, so the presented features are not ordered by their importance.

#### 3.2.3. Gradient Boosting Classifier

**Figure 2:**
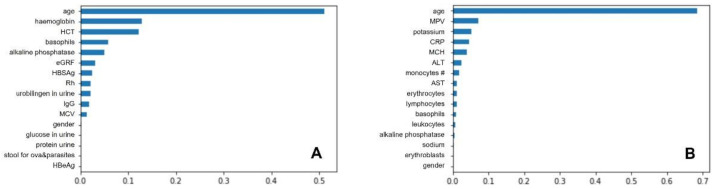
Gradient boosting classifier features importance for ulcerative colitis (**A**) and Crohn’s disease (**B**). Abbreviations for (**A**): HCT, hematocrit; eGFR, estimated glomerular filtration rate; HBsAg, Hepatitis B s Antigen; IgG, immunoglobulin G; MCV, mean corpuscular volume; HBeAg, Hepatitis B e Antigen; Abbreviations (**B**): MPV, mean platelet volume; CRP, C-reactive protein; MCH, mean corpuscular haemoglobin; ALT, alanine aminotransferase; AST, aspartate aminotransferase.

The GBC algorithms were tuned using coefficients such as the number of reinforcement steps to be performed, the penalty function, the learning rate, or the quality measure function of the division. The first optimal value discovered was the exponential penalty function. Changing the penalty function to the exponential function showed an improvement in prediction performance for all quality measurement functions. A similar effect was obtained by changing the learning rate, whose both increased and decreased value positively affected the prediction quality. However, increasing this parameter by one order of magnitude gave better results than decreasing it, so a value of 0.1 was taken as the optimal learning rate value. The expected result was an improvement in prediction for increasing the number of reinforcement steps, whose optimal value was set at 200. As for the criteria for measuring the quality of the partitioning, both had similar results, slightly better for the Friedman mean square error (MSE) criterion. Finally, the following hyperparameters were found to be the optimal for UC dataset: {‘classifier__C’: 1.0, ‘classifier__kernel’: ‘linear’, ‘featureSelector__n_features_to_select’: 19}, and the most relevant were following features: age, haemoglobin, HCT, basophils, alkaline phosphatase, eGRF, HBSAg, Rh, urobilinogen in urine, IgG, MCV, gender, glucose in urine, protein in urine, stool for ova&parasites, HBeAg. Meanwhile for CD dataset the most optimal clasificator parameters were {‘classifier__C’: 0.1, ‘classifier__kernel’: ‘linear’, ‘featureSelector__n_features_to_select’: 20}, resulting in the following important features: age, MPV, potassium, CRP, MCH, ALT, monocytes count, AST, erythrocytes, lymphocytes, basophils, leukocytes, alkaline phosphatase, sodium, erythroblasts, and gender. Gradient boosting classifier features importance were presented in Figure 2.

#### 3.2.4. Random Forests

Random forests perform very well with these types of data for classification, so more than 70 combinations of parameters such as maximum tree depth, number of trees in the forest, number of features considered for partitioning, partition quality measurement function, or bootstrap were carefully checked.

**Figure 3:**
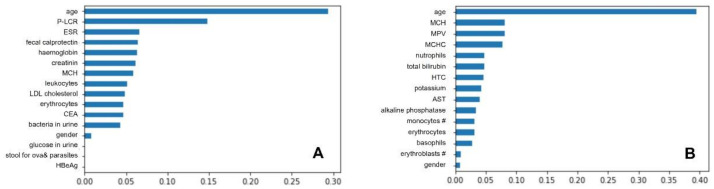
Random forests features of importance for ulcerative colitis (**A**) and Crohn’s disease (**B**). Abbreviations for (**A**): P-LCR, Platelet larger cell ratio; ESR, erythrocyte sedimentation rate; HGB, haemoglobin, MCH, mean corpuscular haemoglobin; LDL, low density lipoprotein; CEA, carcinoembryonic antigen; HBe Ag, Hepatitis B e Antigen; Abbreviations (**B**): MCH, mean corpuscular haemoglobin, MPV, mean platelet volume, MCHC, mean corpuscular haemoglobin concentration, HCT, hematocrit, AST, aspartate aminotransferase, AP, alkaline phosphatase.

All created trees had good prediction results, which showed a good fit to the dataset. For decision trees, pruning is very important, so different spanning-tree values were carefully tested. The best results were characterized by trees with five and three nodes. The value of three nodes was considered as the optimal value, as they received slightly better results, and makes the model less complicated. Increasing the number of trees in the forest had a similar effect; the more trees, the better the prediction obtained. About 200 trees in the forest were considered the optimal value. In contrast, bootstrap did not change the prediction significantly and there were very slight differences, as with the number of features considered for partitioning. For bootstrap, ‘True’ was considered the optimal value, and for the number of features considered this value is the one chosen automatically. Changing the function of measuring the quality of division to entropy improved the quality of prediction, so this was also considered the optimal value. Finally, the following hyperparameters were found to be the optimal for UC dataset: {‘classifier__n_estimators’: 1000, ‘featureSelector__n_features_to_select’: 16}, and the most relevant were following features: age, platelet larger cell ratio (P-LCR), erythrocyte sedimentation rate (ESR), fecal calprotectin(FC), HGB, creatinine, mean corpuscular haemoglobin (MCH), leucocytes, cholesterol-low-density lipoprotein, erythrocytes, carcinoembryonic antigen (CEA), bacteria in urine, gender, glucose in urine, microscopic stool ova and parasites test, and HBe Ag. Meanwhile for CD dataset the most optimal clasificator parameters were {‘classifier__n_estimators’: 1000, ‘featureSelector__n_features_to_select’: 15}, resulting in the following important features: Age, mean corpuscular haemoglobin (MCH), MPV, MCHC, neutrophils, total bilirubin, hematocrit (HCT), potassium, AST, alkaline phosphatase, monocytes, erythrocytes, basophils, and erythroblasts. Random forest features of importance are presented in Figure 3.

#### 3.2.5. Support Vector Classifiers

Properly tuned SVC models had the best predictions of all classifiers. Different combinations of kernel types and their coefficient, independent kernel parameter for specific kernel types, regularization parameter, and degree for polynomial kernel were checked to achieve the optimal solution.

The basic parameter to be checked is the type of kernel. Three types of the kernel can be considered as optimal: RBF, linear, and polynomial kernel. Each of them is characterized by a different set of kernel coefficients, so the same results were obtained only after proper tuning. Changing the gamma coefficient, which is the coefficient for RBF and polynomial kernels, did not indicate significant changes, so both the default value and the automatic value were considered optimal values. For the polynomial kernel, the important coefficients were the polynomial degree and the independent parameter of the kernel. The default degree of the polynomial kernel is degree three, but decreasing this degree resulted in better prediction results. However, tuning other parameters resulted in equally good results of this degree, so the third and first degree of the polynomial (which is formally a linear function), were considered optimal values. Kernels with a polynomial degree of three had better results for the independent degree of the polynomial, which value was set to one. However, for linear solutions, this factor did not matter, and then this value was set to the default value. Changing the penalty regularization coefficient, on the other hand, resulted in worse prediction quality when it was decreased, but an improvement was observed when it was increased. A value of 2.5 was considered the optimal value for the penalty regularization coefficient.

**Figure 4:**
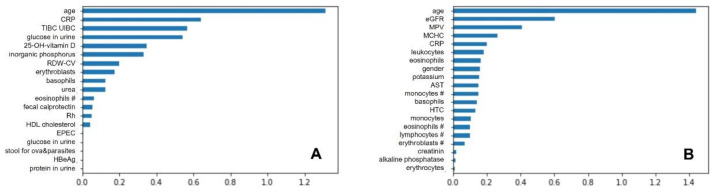
SVC features importance for ulcerative colitis (**A**) and Crohn’s disease (**B**). Abbreviations for (**A**): CRP, C-reactive protein; TIBC, total iron binding capacity; UIBC, unsaturated iron binding capacity; HDL, high-density lipoprotein; Abbreviations (**B**): eGFR, estimated glomerular filtration rate; MPV, mean platelet volume; MCHC, mean corpuscular haemoglobin concentration; CRP, C-reactive protein; AST, aspartate aminotransferase.

It is important to mention here that the *permutation feature importance* algorithm is an algorithm that indicates the relevance of an attribute in a data frame for supervised learning. This algorithm defines attribute relevance as the increase in model prediction error when a single attribute is randomly shuffled. This procedure breaks the link between conditional and decision attributes. Namely, the decrease in the prediction score indicates how dependent the model is on the attribute. An internal parameter called *n_repeats* can be defined for this algorithm, which defines the number of random shuffles. Finally, the following hyperparameters were found to be the optimal for UC dataset: {‘classifier__C’: 1.0, ‘classifier__kernel’: ‘linear’, ‘featureSelector__n_features_to_select’: 19}, and the most relevant were following features: age, CRP, TIBC, UIBC, glucose in urine, 25-OH-vitamin D, inorganic phosphorus, RDW-CV, erythroblasts, basophils, urea, eosinophils count, fecal calprotectin, Rh, HDL cholesterol, EPEC, glucose in urine, stool for ova&parasites, HBeAg, and protein in urine. Meanwhile for CD dataset the most optimal clasificator parameters were {‘classifier__C’: 0.1, ‘classifier__kernel’: ‘linear’, ‘featureSelector_ _n_features_to_select’: 20}, resulting in the following important features: age, eGFR, MPV, MCHC, CRP, leukocytes, eosinophils, gender, potassium, AST, monocytes count, basophils, HTC, monocytes, eosinophils count, lymphocytes count, erythroblasts count, creatinine, alkaline phosphatase, and erythrocytes. SVC features importance were presented in Figure 4.

#### 3.2.6. Majority Voting

Majority voting is meaningful since each case is considered by multiple classifiers, allowing for a good prediction even if one classifier stops handling the data. Of course, one should also not put too many classifiers, since then the chance that a large number of models will lead to voting for incorrect results increases. In the current case, majority voting was at the level of the best single classifier and did not introduce any significant improvement in prediction performance.

### 3.3. Best Classifiers and Most Important Predictors

The initial prediction was made for 64 features, shown in Table 3 which means that a high prediction score can be achieved for a patient with 62 tests performed and age and gender completed. This is not an economical solution from the GP’s point of view. Therefore, cut-off thresholds between 10 and 20 features of significance were introduced. This means that the prediction accuracy was checked for 20, 19, 18, …, 10 features, respectively. The prediction improved for the 15 or 16 cut-off thresholds, but decreased again for the lower and higher number of important features. This may indicate a negative effect of insignificant features on prediction quality, but also a negative effect of too few features. The more insignificant features we remove, the better the prediction for the disease entity and the smaller the standard deviation. However, the removal should not be exaggerated, as too large a cutoff may lead to a deterioration in model quality. Finally, for the UC case, 16 features resulted as an optimal value, while for the CD case 15 features the best optimized prediction quality of the model. Both models were based on random forests classifier.

**Figure 5:**
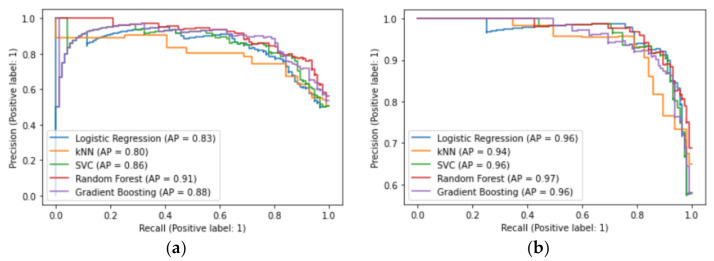
Precision-recall curves for trained classifiers on UC (**a**) and CD (**b**) datasets. Abbreviations: kNN, k-nearest neighbor; SVC, support vector classifiers; AP, average precision.

Based on UC dataset compared to control this best classifier achieved the mean Average Precision (mAP) level of 91%. Meanwhile, for the CD dataset the best classifier obtained the mAP level of 97%. Both were tested by a cross-validation method. Precise model metrics are presented in Table 4 and Table 5 and Figure 5. Average Precision values were calculated as the area under the precision-recall curves shown in Figure 5, and directly refers to information of how many of the predictions were correct, for the given model. Thus, one of the most informative ways to present the robustness of trained classifiers is the precision-recall curve that shows the trade-off between precision and recall for different thresholds. Expecting the high area under the curve one can gain both high precision and high recall, which is not obvious to obtain. In this case a high precision refers to a low false positive rate, while a high recall corresponds to a low false negative rate, which is the most expected situation. Here we can assume that the random forest classifier chosen as the best one, also provides a reasonable precision-recall trade-off.

Another important metric is the recall itself (see Table 4 and Table 5) referring to how well you find all the positives, and presenting directly a ratio of True Positives (predicted as positive as was correct) over True Positives + False Negatives (failed to predict an object that was there). As opposed to AP, in this metric one can quantify the number of correct positive predictions made out of all possible positive predictions, indicating missed positive predictions.

### 3.4. Model Robustness 

The most significant feature appeared to be patient age which may be a direct result of control group selection. We tried to get as close as possible to a control group with similar symptoms but different diagnosis, and free of leading comorbidities. It is no secret that the level of prediction strongly depends on the selection of the control group, and with the introduction of additional diseases occurring in younger patients, this feature may cease to be important and the quality of prediction may deteriorate. Based on the assumption of the validity of selecting such a control group, we ran a test removing this feature from the dataset, and to our surprise we obtained predictions accuracy at the same level but with a deviation that was on average 2% larger. 

It is also important to remember that the extracted data are not immune to physician error, who may have recorded the wrong value, or to coarse errors related to the nature of the biological data and the special case of a patient who has results higher than others.

Real data as input to a learning algorithm are also subject to noise. Noise refers to additional unwanted elements in the original data set that disrupt the relationship between an object attribute and its class. Noise can directly affect performance in terms of accuracy, model size, and time to build a machine learning model, and cause prediction errors. The presence of noise in the data results in distortions that can introduce new properties in the problem domain. A quantitative study of noise on classification algorithms has shown that as attribute noise increases, the accuracy of classification algorithms decreases linearly at a ratio of 1% accuracy per 1% noise (in the range of up to 10% noise). This linear dependency is coherent with the algorithm documentation. However, our approach through the random forest algorithm showed particular robustness to noise by oscillating around the original accuracy value only by ±1% (again in the range up to 10% of the noise).

### 3.5. Comparison of the Machine Learning Model to C-Reactive Protein in the Prediction of the IBD Presence

**Figure 6:**
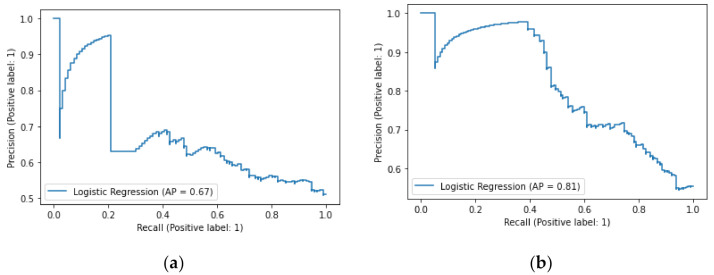
Precision-recall curves for trained classifiers on UC (**a**) and CD (**b**) datasets, basing on CRP level only. Abbreviations: AP, average precision; CRP, C-reactive protein.

We decided to compare our model to the standard inflammatory serum marker, that is CRP. Since training the random forest model with one parameter only (CRP level) misses the assumptions of this algorithm, we prepared the linear regression models for both UC and CD separately, similar to the description made above in Section 3.2.1. Models with a linear optimization received poor to average results, as expected. The following hyperparameters were found to be the optimal for UC dataset: {‘classifier__C’: 0.01, ‘classifier__penalty’: ‘l2’, ‘classifier__solver’: ‘newton-cg’}. For the CD case the best hyperparameters were as follows: {‘classifier__C’: 10.0, ‘classifier__penalty’: ‘l2’, ‘classifier__solver’: ‘newton-cg’}. Machine learning model optimized only on CRP level provides a very poor prediction for ulcerative colitis, which is expressed in low average precision of 67%, and positively found true cases of disease incidence, with recall metric of only 34%. Crohn’s disease CRP level-based prediction has better performance than in the UC case, but still with average ability. The linear regression model for the CD presents average precision on a good enough 81% level, but at the same time recall metric shows only 56%, which means that the model can identify only half of the existing cases of the disease. All details are provided for comparison in Table 6 and Table 7 and Figure 6.

### 3.6. Web Application Integrated Model

We encourage mainly primary care physicians but also other health care professionals to test our ML prediction model at the early stage of the diagnostic workup. Separate machine learning (ML) models that have been trained for both discussed here IBD entities can be tested on the following website: 

https://ml-for-bowel-disease.herokuapp.com/ accessed on 9 August 2021.

We inform all readers that our predictive model does not constitute novel nor substitutive approved medical procedure in IBD diagnosis or even alternative approach substituting colonoscopy, and has not received any approval from any health agency such as the Food and Drug Administration (FDA) or European Medicines Agency (EMA). We present our ML model for educational purposes only, and any use is at your own risk and cannot give rise to any claims.

## 4. Discussion

We demonstrated that machine learning prediction model based on simple, available, and broadly applied blood, urine, and fecal biomarkers as well as age of patient may (with high accuracy) support diagnosis of both ulcerative colitis and Crohn’s disease.

Considering the complexity of every aspect of IBD, from pathogenesis to therapy, artificial intelligence (AI) is a promising approach to expand the knowledge on IBD etiology, support diagnosing and stratification of IBD patients as well as implementation of precision medicine into clinical practice [13]. Further, AI-endoscopy and especially AI-driven endoscopy in IBD, although still requiring further research, holds a great promise in improving IBD assessment [14,15]. Moreover, it was demonstrated that machine learning methods may be applied to identify risk of therapy complications in IBD. For instance, McDonell et al. demonstrated that random forest (RF) regression models identified higher levels of CRP and longer duration of disease as predictors for hyperglycemia in IBD patients treated with intravenous glucocorticosteroids [16]. In turn, Choi et al. developed a machine learning model to predict the five-year risk of starting biologic therapy in IBD patients [17].

Nevertheless, AI in IBD is usually employed for translational research and IBD diagnosis based on microbiome analysis [18]. For instance, machine learning model, specifically random forest, yielded high accuracy (area under the curve, (AUC) > 0.9) in prediction of IBD using 117 differential bacterial taxa [19], whereas Khorasani et al. developed a model based on the expression of 32 genes expressed in colon samples [20].

**Figure 7:**
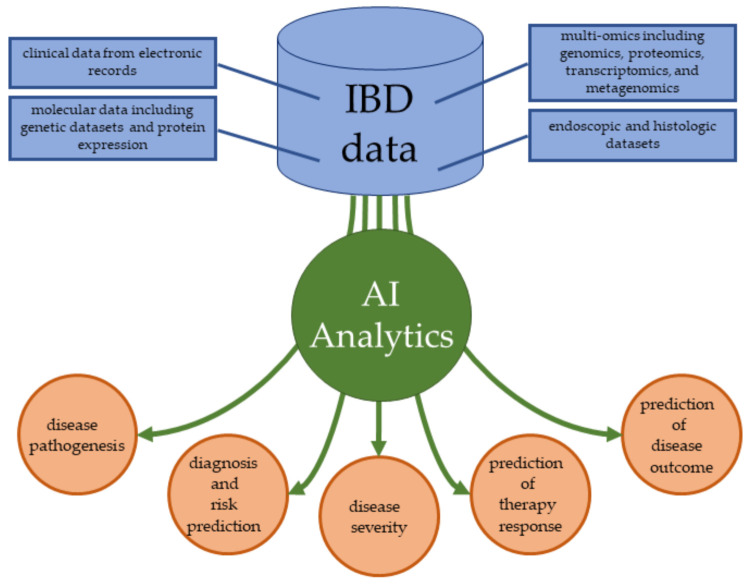
Location of artificial intelligence (AI) in the medical management of inflammatory bowel disease as proposed by Gubatan et al. [21]. IBD, inflammatory bowel disease.

A broad spectrum of applications of AI in inflammatory bowel disease was also reviewed by Gubatan et al. [21] as we demonstrated in Figure 7.

In turn, we took an attempt to analyse with machine learning models simple laboratory tests performed in real clinical life. Most robust classificators belonging to the random forest family obtained 97% and 91% mean average precision for Crohn’s disease and ulcerative colitis, respectively. The feasibility of making UC diagnoses using non-invasive methods is possible by the random forest classifier we selected, which achieved satisfactory results, when matching to age, gender, and 14 laboratory features that can be easily verified by a general practitioner: P-LCR, ESR, fecal calprotectin, haemoglobin, creatinine, MCH, peripheral blood leukocytes, cholesterol-LDL, peripheral blood erythrocytes, CEA, bacteria in urine, glucose in urine, microscopic stool ova and parasites test, and HBeAg. In turn for CD, the random forest algorithm showed that the most significant attributes were age, gender, and further laboratory markers: MCH, MPV, MCHC, peripheral blood neutrophils, total bilirubin, HCT, potassium, AST, AP, peripheral blood monocytes, erythrocytes, basophils, and erythroblasts. 

It has to be emphasized that both, developed model and web application, require further research in the large cohort of patients with suspicion of IBD and comparison to endoscopy as a reference test.

Multiple biomarkers, which have been identified in presented models as important disease descriptors, have pathogenetic connection with the inflammatory bowel disease, mainly in respect to indices of inflammation, anemia, and malnutrition.

First of all, ESR and CRP represent classic serum inflammatory markers [22], whereas fecal calprotectin reflects gastrointestinal inflammation [23]. CRP is an acute-phase reactant produced by hepatocytes in response to stimulation from inflammatory cytokines such as interleukin-1, interleukin-1β, and tumor necrosis factor-alpha [24], whereas, ESR indicates the migration speed of red blood cells in plasma [25] Meta-analysis aimed to assess the utility of CRP, ESR, FC, and fecal lactoferrin to exclude inflammatory bowel disease in adults with IBS demonstrated that at a CRP level of ≤0.5 or calprotectin level of ≤40 μg/g, there was a ≤1% probability of having IBD. However, individual analysis of ESR had little clinical utility [26]. It has to be emphasized that measurement of fecal calprotectin is a non-invasive test that has an established position in the differentiation between inflammatory and non-inflammatory gastrointestinal conditions, for instance, irritable bowel syndrome (IBS) as well as monitoring of the IBD course [27]. 

Yet, the assessment of fecal calprotectin concentration has also several limitations. For instance, it may be influenced by:

- gastrointestinal infections, gastric and colonic malignancies, eosinophilic colitis, lymphocytic colitis, and coeliac disease,
- concomitant medical treatment with proton pump inhibitors, nonsteroidal anti-inflammatory drugs, and acetylsalicylic acid,
- age.

Other limitations of FC are related to substantial inter-assay differences between FC tests from different manufacturers and daily variability of FC concentrations in stool [7], thus we decided to compare our ML model to CRP-based diagnosis only.

Among identified parameters, a complete blood count is a standard laboratory test performed during initial evaluation of patients suspected to have IBD. Multiple parameters related with platelets (P-LCR; MPV), hemoglobin (MCH; MCHC) as well as hematocrit, leukocytes and erythrocytes counts were already known as robust disease descriptors. Abnormalities in complete blood count in IBD correspond mainly with anemia, inflammation, iron and vitamins deficiencies, or applied previous therapy. Furthermore, iron and ferritin were also incorporated in our ML algorithm. Aberrations in their serum concentration of iron and ferritin have complex background and are associated primarily with gastrointestinal bleeding and malabsorption. Moreover, lower iron and ferritin concentration were found to be present in IBD patients long before the establishment of the diagnosis [28]. Furthermore, cholesterol is accounted for, in the indices of malnutrition [29]. Similarly, deficiencies of vitamin D are common in IBD patients, especially in CD cases. However, all above mentioned parameters handled individually are far from being perfect diagnostic markers in IBD [30]. They are often used in clinical practice as supplementary examinations in combination with other laboratory and imaging tests, mainly colonoscopy. However, among the recurring disease descriptors in less predictive classifiers we found surprisingly also aminotransferases and GGTP, which are not routinely used as diagnostic markers in IBD. Serum aminotransferase elevation in IBD may be triggered by several factors, for instance by immunosuppressive therapy [31] as well as liver and biliary tract IBD-related comorbidities. Cholestatic biochemical profile with elevated gamma-glutamyl transpeptidase (GGTP) related with elevated transaminase levels were found in pediatric study concluded that abnormal liver enzymes with elevated GGTP levels (>252 U/L), were commonly found in pediatric IBD with associated primary sclerosing cholangitis (PSC) [32]. Based on all here studied classificators, we postulate that even slightly elevated GGTP levels may be associated with at least specific IBD course, and merits further study.

Several parameters, like creatinine, CEA, or urine test findings do not have a direct pathogenetic link with IBD and the presence of them in the model cannot be easily explained. Nevertheless, intentionally we did not limit our study to the well-known IBD-related markers, and these parameters influenced the effectiveness of our model. Further, prospective cohort study can address their role.

It was previously demonstrated that the accuracy of identifying IBD may be reached by applying the combination of markers [33]. The incorporation of multiple markers to the ML models may decide about their advantages over single tests. Comparing our many-parameter model, with CRP we found out that at most, based on CRP level one can identify only a half of the IBD cases. This is valid for the CD case where the recall parameter reaches 56%, whereas for ulcerative colitis this confidence extends to only one-third of actual cases (recall of 34%). Following this reasoning, it is far from being enough in the diagnostic processes. Presented here the random forest classifiers reach the general recognition of actual cases of UC at 89%, and CD at 95% levels. All concatenated metrics of the random forest models result thus in average precision of prediction with 91% (UC) and 97% (CD) values. Compared to the CRP level, we double or even triple the prediction accuracy reaching the acceptable level. 

Moreover, as age can influence the results of FC, a key non-invasive marker of gastrointestinal inflammation, including the age into our ML algorithm might be also beneficial, and in the presented model appear even more related than FC. For illustration, FC sensitivity in differentiation between controls and IBD patients was satisfactory (68%), and the specificity was high (93%) among young (<65 years), but not among older (≥65 years) subjects [34]. Therefore, the proposed model can be particularly beneficial in older patients, yet, further studies are obviously needed.

Laboratory markers identified by our machine learning algorithms may be seen, at least partly, as unexpected. One would rather presume that the parameters identified by AI will be limited to the classical laboratory tests employed in IBD diagnostics. Indeed, our model incorporates classical markers but also several others, which were unforeseen, and their importance is not obvious. Yet, we applied per purpose all laboratory tests performed on our patients to give the chance to AI to determine which can be useful in such a clinical situation. We also did not include symptoms in the algorithm as we decided to rely only on measurable, and therefore objective parameters. It has to be highlighted that the ML algorithm combines several parameters and interprets the interrelation between them. Moreover, the inclusion of selected parameters into the ML model may not be explainable directly, since there is a chance that some of those parameters are only close enough derivatives of other parameters, that would better identify IBD presence, but were not present in our dataset. Thus one should not unreflectively rely on proposed here descriptors, but again our findings necessitate further clinical investigation. More precisely, the standard approach to the assessment of single markers, like CRP or FC, relies on the correlation between their concentrations and inflammation. Contrary, the ML model predicts the presence of disease based on the existence and/or values of all studied parameters at a certain point in time. Therefore, based on our algorithm it cannot be concluded, that for instance the younger or older age and lower or higher hemoglobin, etc. are separate predictors of IBD as the parameters in the ML model are not to be considered individually. The one we have shown is that the many-parameters ML model is far more effective in the prediction of IBD presence than CRP. Our model was trained to predict only the presence or absence of IBD disease. It was not created to indicate disease extent or severity, therefore these features were not included in the analysis.

We are aware that our research has several limitations. First of all, we included in our study both, patients with newly established IBD diagnosis as well as patients which have been already previously diagnosed with IBD. Therefore, we cannot exclude that disease duration, past and present therapy as well as extraintestinal manifestations had influenced results of analyzed laboratory tests. On the other hand, complicated clinical manifestations of IBD together with challenging diagnostic algorithms are responsible for the diagnostic delay. Hence, every tool which can support an early diagnosis is valuable. Further, age in our algorithms turned out to be a factor helping in the differentiation between IBD and non-IBD patients, which resulted from the age profile of the control group. Ideally, the control group should be chosen from the patients at similar age such as the studied group. However, as our study was hospital based it was impossible to construct a control group with enough young patients without inflammatory disease, as such patients are usually diagnosed in an ambulatory way. Moreover, the age profile of IBD patients is changing due to growing incidence of early- and late-onset IBD cases [35]. Another limitation of the present study is the fact that most of the samples in all groups were not complete. We addressed this issue with data supplementation by multiple imputation method using IterativeImputer from the Scikit-learn library as described in the Methods section. 

Finally, it has to be emphasized that the test can’t be seen as sufficiently valid, and prospective cohort study has a particular meaning in the evaluation of its effectiveness. The current form has only an informative form and cannot be applied in clinical practice.

## 5. Conclusions

Complex and unclear pathogenesis of inflammatory bowel disease stays behind the failure into identifying a single biomarker of disease. As IBD has become a global disease, simple and available diagnostic tools are essential. Therefore, we attempted to create a machine-learning algorithm to support IBD diagnosis. Results of our pilot study suggest that routine blood, urine and fecal markers based machine learning model may support with high accuracy, higher than CRP, the diagnosis of IBD. However, validation of the test is vital before it can be considered to be applied in clinical practice.

## Figures and Tables

**Table 1:** Characteristics of ulcerative colitis and Crohn’s disease patients according to the Montreal classification.

Characteristic	Ulcerative Colitis *N* = 319	Crohn’s Disease *N* = 383
Age at diagnosis, y (%)		
A1, below 16 years		38 (9.9)
A2, between 17 and 40 years		234 (51.1)
A3, above 40 years;		11 (29.0)
Behaviour, *n* (%)		
B1, non-stricturing, non-penetrating		298 (77.8)
B2, structuring		57 (14.9)
B3, penetrating		28 (7.3)
Extent, *n* (%)		
E1, proctitis	149 (46.7)	
E2, left-sided colitis	100 (31.3)	
E3, extended colitis	E3 70 (22)	
Location, *n* (%)		
L1, ileal		85 (22.2)
L2, colonic		107 (27.9)
L3, ileocolonic		191 (49.9)
Severity, *n* (%)		
S0, clinical remission	162 (50.8)	
S1, mild ulcerative colitis	39 (12.2)	
S2, moderate ulcerative colitis	70 (22)	
S3, severe ulcerative colitis	48 (15.1)	

y, years; *n*, number;.

**Table 2:** Baseline physical examination and laboratory characteristics.

Characteristic	Ulcerative Colitis*N* = 319	Crohn’s Disease *N* = 383	Control *N* = 315
Age, y	41.8 ± 14.7	36.3 ± 12.6	63.8 ± 13.4
Female gender, *n* (%)	183 (57)	159 (41)	135 (43)
C-reactive protein level, mg/L	20.7 ± 50.8	25.1 ± 41.7	5.5 ± 15.4
Sodium, mmol/L	139.2 ± 2.9	139.3 ± 2.3	140.0 ± 2.5
Potassium, mmol/L	4.1 ± 0.4	4.3 ± 0.3	4.3 ± 0.5
eGFR, mg/dL	90.5 ± 18.1	214.9 ± 526.8	76.7 ± 19.5
Random blood glucose, mg/dL	104.9 ± 31.1	96.5 ± 11.0	108.4 ± 30.8
Complete blood count			
White blood cell, 103/µL	8.1 ± 3.5	7.5 ± 2.6	6.8 ± 1.9
Hemoglobin, g/dL	12.6 ± 2.1	12.6 ± 1.9	13.7 ± 1.8
Hematocrit, %	38.6 ± 5.3	38.6 ± 4.7	41.0 ± 4.8
Activated partial thromboplastin time, sec.	27.7 ± 6.0	27.3 ± 3.4	26.3 ± 3.8
Liver function test			
Alanine transaminase, U/L	32.5 ± 43.0	20.8 ± 15.5	27.0 ± 15.1
Aspartate transaminase, U/L	32.4 ± 35.6	23.4 ± 9.4	30.5 ± 17.1
Alkaline phosphatase, U/L	124.9 ± 174.7	86.1 ± 36.4	82.1 ± 43.5
Total bilirubin, mg/dL	0.87 ± 0.99	0.71 ± 0.48	0.96 ± 1.27

y, years; *n*, number; mmol/L, millimoles per litre; mg/dL, milligrams per decilitre; 10^3^/µL, kilo per microliter; U/L, Units/Litre; sec., seconds.

**Table 3:** Filtered input features in alphabetical order used for the machine learning model.

Numerical Value *	Converted Text Value ^1^	Binary Value *
25-OH-Vitamin D	bilirubin in urine	disease diagnosis ^2^
age	blood in urine	gender ^3^
ALT	ketones in urine	HBeAg
alkaline phosphatase	leukocytes in urine	stool ova and parasites microscopic test
APTT	nitrites in urine protein in urine	
AST	squamous epithelial cells in urine	
basophils %		
basophils #		
bilirubin (total)		
cholesterol (HDL)		
cholesterol (LDL)		
cholesterol (total)		
eGFR		
eosinophils %		
eosinophils #		
erythroblasts %		
erythroblasts #		
erythrocytes %		
Ferritin fecal calprotectin		
folic acid		
GGTP		
glucose		
haemoglobin level haemoglobin A1c		
HTC %		
immature granulocytes # ^4^		
iron		
leukocytes %		
lipase		
lymphocytes %		
lymphocytes #		
MCH		
MCHC		
MCV		
monocytes %		
monocytes #		
MPV		
neutrophils %		
neutrophils # PCT		
potassium		
protein (total)		
PT (index)		
PT/INR		
amylase		
creatinine		
sodium		
TSH 3rd generation		
ultra-sensitive CRP		
urine pH		
urobilinogen in urine		
vitamin B12		

^1^ 0-uncommon, 1-trace, 2-individual, 3-quite frequent, 4-numerous, 5-abundant. ^2^ 0-nonexistent, 1-existent. ^3^ 0-female, 1-male. ^4^ Immature granulocytes, i.e., meta-, myelo-, and promyelocytes. * if not stated different tests are blood-based. #, count; %, percentage; ALT, alanine aminotransferase; AST, aspartate aminotransferase; APTT, activated partial thromboplastin time; CRP, C-reactive protein; eGFR, estimated glomerular filtration rate; GGTP, gamma-glutamyl transpeptidase; HCT, hematocrit; HBeAg, Hepatitis B e Antigen; HDL, high-density lipoprotein; INR, international normalized ratio; LDL, low-density lipoprotein; MCH, mean corpuscular haemoglobin; MCV, mean corpuscular volume; MCHC, mean corpuscular haemoglobin concentration; MPV, mean platelet volume; PT, prothrombin time; PCT, plateletcrit; TSH, thyroid-stimulating hormone.

**Table 4:** Metrics for the random forest Machine Learning model for UC prediction.

	Precision	Recall	F1-Score	Support
no disease (0)	0.86	0.73	0.79	94
UC present (1)	0.77	0.89	0.83	96
accuracy	-	-	0.81	190
macro average	0.82	0.81	0.81	190
weighted average	0.82	0.81	0.81	190

UC, ulcerative colitis.

**Table 5:** Metrics for the random forest Machine Learning model for CD prediction.

	Precision	Recall	F1-Score	Support
no disease (0)	0.92	0.76	0.83	94
CD present (1)	0.83	0.95	0.88	115
accuracy	-	-	0.86	209
macro average	0.87	0.85	0.86	209
weighted average	0.87	0.86	0.86	209

CD, Crohn’s disease.

**Table 6:** Metrics for the CRP level-based model for UC prediction.

	Precision	Recall	F1-Score	Support
no disease (0)	0.55	0.82	0.66	94
UC present (1)	0.66	0.34	0.45	96
accuracy	-	-	0.58	190
macro average	0.60	0.58	0.56	190
weighted average	0.61	0.58	0.55	190

UC, ulcerative colitis; CRP, C-reactive protein.

**Table 7:** Metrics for the CRP level-based model for CD prediction.

	Precision	Recall	F1-Score	Support
no disease (0)	0.59	0.77	0.66	94
CD present (1)	0.74	0.56	0.64	115
accuracy	-	-	0.65	209
macro average	0.66	0.66	0.65	209
weighted average	0.67	0.65	0.65	209

CD, Crohn’s disease; CRP, C-reactive protein.

## Data Availability

The data presented in this study are available on request from the corresponding author. Pretrained machine learning models are available for educational purposes only on the following website: https://ml-for-bowel-disease.herokuapp.com/ (accessed on 15 October 2021).
